# Phase Separation Drives Pathological Aggregation in Neurodegenerative Diseases: A 15‐Year Bibliometric Landscape (2009–2024)

**DOI:** 10.1111/nyas.70311

**Published:** 2026-07-08

**Authors:** Chanyuan Zhang, Siyao Chen, He Zhao, Yan Wang, Lingyan Zhou, Haixia Fan, Yan Sun

**Affiliations:** ^1^ Department of Otorhinolaryngology, Head and Neck Surgery, Yantai Yuhuangding Hospital Qingdao University Yantai Shandong China; ^2^ Shandong Provincial Key Laboratory of Neuroimmune Interaction and Regulation Yantai Shandong China; ^3^ Shandong Provincial Clinical Research Center for Otorhinolaryngologic Diseases Yantai Shandong China; ^4^ Yantai Key Laboratory of Otorhinolaryngologic Diseases Yantai Shandong China; ^5^ Department of Sleep Center First Hospital of Shanxi Medical University Taiyuan Shanxi Province China; ^6^ Department of Neurology Shandong Provincial Hospital Affiliated to Shandong First Medical University Jinan Shandong China

**Keywords:** Alzheimer's disease, bibliometric analysis, liquid–liquid phase separation, neurodegenerative diseases, pathological aggregation

## Abstract

Liquid–liquid phase separation (LLPS), a biophysical driver of membraneless organelle assembly, is central to pathological aggregation in neurodegenerative diseases. Initially linked to amyotrophic lateral sclerosis (ALS), LLPS dysregulation has now been implicated in Alzheimer's, Parkinson's, and frontotemporal dementia, where aberrant transitions convert dynamic condensates into insoluble fibrils. To systematically map this landscape, we employed CiteSpace‐based bibliometrics to analyze 784 Web of Science articles from 2009 to 2024. Our analyses reveal dominant contributions from the United States, China, and Germany, with collaborative networks focusing on protein dynamics. Key hotspots include LLPS‐driven aggregation of TARDBP (TDP‐43), FUS, and α‐synuclein, alongside stress granule dysfunction and nucleocytoplasmic transport defects. Emerging frontiers highlight therapeutic strategies targeting pathological condensates utilizing small‐molecule chaperones and posttranslational modification modulators to restore cellular homeostasis. Our findings underscore LLPS as a critical axis bridging molecular pathology and translational innovation. The field is rapidly shifting from mechanistic exploration to therapeutic applications, emphasizing interventions to halt or reverse aggregation. By delineating global trends and changing priorities, our study highlights the transformative potential of phase‐targeted interventions and provides a roadmap of groundbreaking interdisciplinary research into neurodegenerative disorders.

## Graphical abstract

This 15‐year bibliometric study (2009–2024) characterizes the global landscape of liquid–liquid phase separation (LLPS) in neurodegenerative diseases. We delineate the field's evolution from fundamental biophysics to clinical translation, emphasizing the liquid‐to‐solid transition of proteins like TDP‐43, tau, and α‐synuclein. Our analysis highlights emerging therapeutic hotspots, including PTMs and molecular chaperones, providing a comprehensive roadmap for next‐generation disease‐modifying interventions targeting condensate homeostasis.

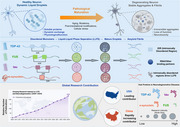

## 1 Introduction

Organelles are crucial for cellular activities and are categorized into membrane‐bound organelles (e.g., endoplasmic reticulum, Golgi apparatus, mitochondria, and lysosomes) and membraneless organelles (MLOs) [1, 2]. The sophisticated crosstalk between membranous and MLOs is beneficial for maintaining homeostasis [3]. Crucially, MLOs provide a dynamic organizational framework for the spatiotemporal compartmentalization of cellular components, regulating diverse biochemical reactions without the need for physical membranes [4]. Before 2009, phase separation was initially considered merely a physicochemical process where molecules segregate into dense and dilute phases [4], and then the studies by Tony Hyman and Cliff Brangwynne on P granules, which helped establish the concept of liquid–liquid phase separation (LLPS) [5, 6] were published. For biomolecules, phase separation exists in the form of liquid droplets [7]. LLPS explains the formation of multiple membraneless cellular structures [8]. It is driven by two types of multivalent interactions: conventional macromolecular interactions (RNA–RNA, protein–RNA, and protein–protein) and transient interactions (π–π, π–cation, cation–anion, and dipole–dipole) in disordered regions [9, 10, 11, 12]. Dynamic multivalent interactions, particularly those involving intrinsically disordered protein regions, are key drivers of LLPS [12, 13, 14]. This emerging mechanism was investigated in the presence, formation, biological functions, and disease associations of MLOs in cells [15, 16]. It is known that LLPS is closely linked to critical cellular processes such as cell fate determination, signal transduction, endocytosis, regulation of gene expression and protein translation, and RNA metabolism [15, 17].

A major turning point in this field was the realization that LLPS is not merely a biophysical phenomenon, but a central mechanism underlying the pathogenesis of diseases [6]. Under physiological conditions, LLPS is highly reversible, allowing MLOs such as stress granules (SGs) to rapidly assemble under cellular stress and disperse once the stress is resolved. However, genetic mutations in RNA‐binding proteins (e.g., TDP‐43 and FUS) or prolonged cellular stress can disrupt this dynamic equilibrium, triggering a shift from a physiological liquid state to a pathological state [18, 19]. This shift manifests as a liquid‐to‐solid phase transition, where highly dynamic liquid condensates gradually mature into irreversible, solid‐like protein aggregates and amyloid fibrils [20]. Furthermore, abnormalities in SGs—such as persistent assembly and impaired clearance—act as crucibles for this pathological aggregation, inexorably linking aberrant phase separation to neurotoxicity [18].

Neurodegenerative diseases (NDDs), such as Alzheimer's disease (AD), Parkinson's disease (PD), and amyotrophic lateral sclerosis (ALS), pose a significant threat to human health and have become a global public health challenge. Since the landmark discovery linking LLPS to TDP‐43 aggregation in ALS [19], LLPS has been implicated in the pathogenesis of AD, PD, and frontotemporal dementia (FTD). Dysregulated phase separation contributes to aberrant protein aggregation—a hallmark of neurodegeneration—by facilitating the transition of dynamic condensates into pathological fibrils [21]. Despite growing recognition of its mechanistic importance, the global research landscape and evolving trends in this interdisciplinary field remain systematically uncharacterized.

Bibliometric analysis has become an important tool for evaluating research trends and identifying hotspots in a specific field [22]. By conducting a bibliometric and visualization study on the research trends of phase separation in NDDs, we aim to provide insights into the intellectual structure and development trends of this research area, which will be helpful for other researchers to better understand the current status and future directions of this field.

This study employs bibliometric analysis using CiteSpace and VOSviewer to map the intellectual architecture of phase separation research in NDDs from 2009 to 2024. Drawing on 784 articles from the Web of Science Core Collection (WoSCC), we conducted co‐authorship analyses of countries/institutions/authors, keyword co‐occurrence networks, and literature co‐citation clustering. Our objectives were threefold: (1) to identify leading contributors (countries, institutions, and authors) driving innovation; (2) to uncover research hotspots such as TDP‐43/FUS/α‐synuclein phase behavior [23] and SG dynamics [24]; and (3) to forecast emerging frontiers, including therapeutic targeting of pathological condensates.

## 2 Methods

### 2.1 Data Sources and Search Strategies

**FIGURE 1:**
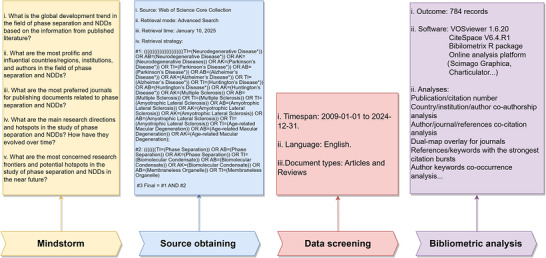
Conceptual design and the workflow diagram. The research design encompasses four sequential phases: (1) Mindstorm: Five core research questions were formulated to investigate global development trends, key contributors (countries/regions, institutions, and authors), preferred journals, evolving research hotspots, and future frontiers regarding phase separation in neurodegenerative diseases (NDDs). (2) Source obtaining: A comprehensive literature retrieval was conducted on January 10, 2025, via the Web of Science Core Collection (WoSCC). The advanced search strategy utilized Boolean operators to intersect terms related to NDDs (e.g., Parkinson's disease, Alzheimer's disease, and ALS) with terms related to phase separation (e.g., biomolecular condensates and membraneless organelles). (3) Data screening: To ensure data quality and relevance, the search was restricted to English‐language original articles and reviews published between January 1, 2009 and December 31, 2024. (4) Bibliometric analysis: Following rigorous screening, a final dataset of 784 valid records was established. Multiple bibliometric tools, including VOSviewer (v1.6.20), CiteSpace (v6.4.R1), the Bibliometrix R package, and online visualization platforms (Scimago Graphica and Charticulator), were systematically employed. Comprehensive evaluations were performed, encompassing publication/citation metrics, co‐authorship networks, co‐citation analysis, dual‐map journal overlays, burst detection for references and keywords, and keyword co‐occurrence networks, to map the knowledge landscape of this field.

Data were sourced from the Web of Science (WoS) Core Collection database (https://www.webofscience.com/wos/woscc/basic‐search). The search index only included SCI‐EXPANDED. All searches and data collection were completed on January 10, 2025, to avoid errors due to database updates. Searches were conducted by topic without language or year restrictions. The search formula used was #1: (((((((((((((((((((((((TI = (Neurodegenerative Disease*)) OR AB = (Neurodegenerative Disease*)) OR AK = (Neurodegenerative Diseases)) OR AK = (Parkinson's Disease*)) OR TI = (Parkinson's Disease*)) OR AB = (Parkinson's Disease*)) OR AB = (Alzheimer's Disease*)) OR AK = (Alzheimer's Disease*)) OR TI = (Alzheimer's Disease*)) OR TI = (Huntington's Disease*)) OR AB = (Huntington's Disease*)) OR AK = (Huntington's Disease*)) OR AK = (Multiple Sclerosis)) OR AB = (Multiple Sclerosis)) OR TI = (Multiple Sclerosis)) OR TI = (Amyotrophic Lateral Sclerosis)) OR AB = (Amyotrophic Lateral Sclerosis)) OR AK = (Amyotrophic Lateral Sclerosis)) OR AK = (Amyotrophic Lateral Sclerosis)) OR AB = (Amyotrophic Lateral Sclerosis)) OR TI = (Amyotrophic Lateral Sclerosis)) OR TI = (Age‐related Macular Degeneration)) OR AB = (Age‐related Macular Degeneration)) OR AK = (Age‐related Macular Degeneration); #2: (((((((TI = (Phase Separation)) OR AB = (Phase Separation)) OR AK = (Phase Separation)) OR TI = (Biomolecular Condensate)) OR AB = (Biomolecular Condensate)) OR AK = (Biomolecular Condensate)) OR AB = (Membraneless Organelle)) OR TI = (Membraneless Organelle); #3 Final = #1 AND #2. A total of 861 documents were retrieved. The conceptual design and flowchart of the study are presented in Figure 1.

### 2.2 Data Screening

The publication types were limited to original articles and reviews, and only literature in English was included. The period of interest was from 2009 to 2024. Any related publications were picked up and saved in plain.txt format for further study; complete records and cited references were also included. The impact factor (IF) and subject category quartile ranks of journals were obtained from the 2024 Journal Citation Reports (JCR). The *h*‐index, as will be discussed below, is defined as the number of papers (*h*) that have received at least *h* citations, serving as a measure of the cumulative impact of a country's research output. For example, an *h*‐index of 20 indicates that 20 documents have each received at least 20 citations.

### 2.3 Bibliometric Analysis

The comprehensive bibliometric and visual analyses were executed using a combination of software and tools: Microsoft Office Excel 2023, CiteSpace 6.4.R1 (Drexel University, PA, United States), VOSviewer 1.6.20 (Leiden University, the Netherlands), the Bibliometrix R‐package in R‐Studio (https://bibliometric.com), and supplementary online visualization platforms including Scimago Graphica (www.graphica.app) and Charticulator (https://ilfat‐galiev.im/charticulator/). VOSviewer 1.6.20 was primarily employed to construct and visualize the collaborative networks of countries, institutions, and authors. To ensure the accuracy of the data, a standardized VOSviewer thesaurus file was utilized to merge synonyms and eliminate variant spellings. During the parameter configuration, “fractional counting” was selected to normalize the weight of multi‐authored documents, reducing the bias caused by publications with extensive author lists. In the generated VOSviewer maps, each node represents a specific entity (e.g., an author, institution, or country), with its size dynamically reflecting the frequency of publications or occurrences. The link lines between nodes illustrate the strength of collaborative relationships. Furthermore, nodes are color‐coded based on the clustering algorithms (the log‐likelihood ratio algorithm) built into VOSviewer, representing different subgroups or research communities. CiteSpace 6.4.R1 Advanced, a Java‐based application designed for visualizing structural and temporal patterns in scientific literature, was utilized for multidimensional network construction. Specifically, it was employed to map subject area distributions, generate keyword timeline views, and detect citation/keyword bursts to identify emerging research frontiers and hotspots over time. To configure the network precisely, the parameters in CiteSpace were established as follows: The time slicing was set from January 2009 to December 2024 with a “Years Per Slice” of 1; the selection criterion was defined using a modified *g*‐index (scaling factor *k* = 25) to extract the most representative items per slice. Finally, Microsoft Excel 2023 was used for basic quantitative trend analyses. The Bibliometrix R‐tool, alongside online platforms like Scimago Graphica, was applied to generate geographical distribution maps and further refine the visual presentation of references and keyword data.

## 3 Results

### 3.1 Annual Trend of Publications and Citations

**FIGURE 2:**
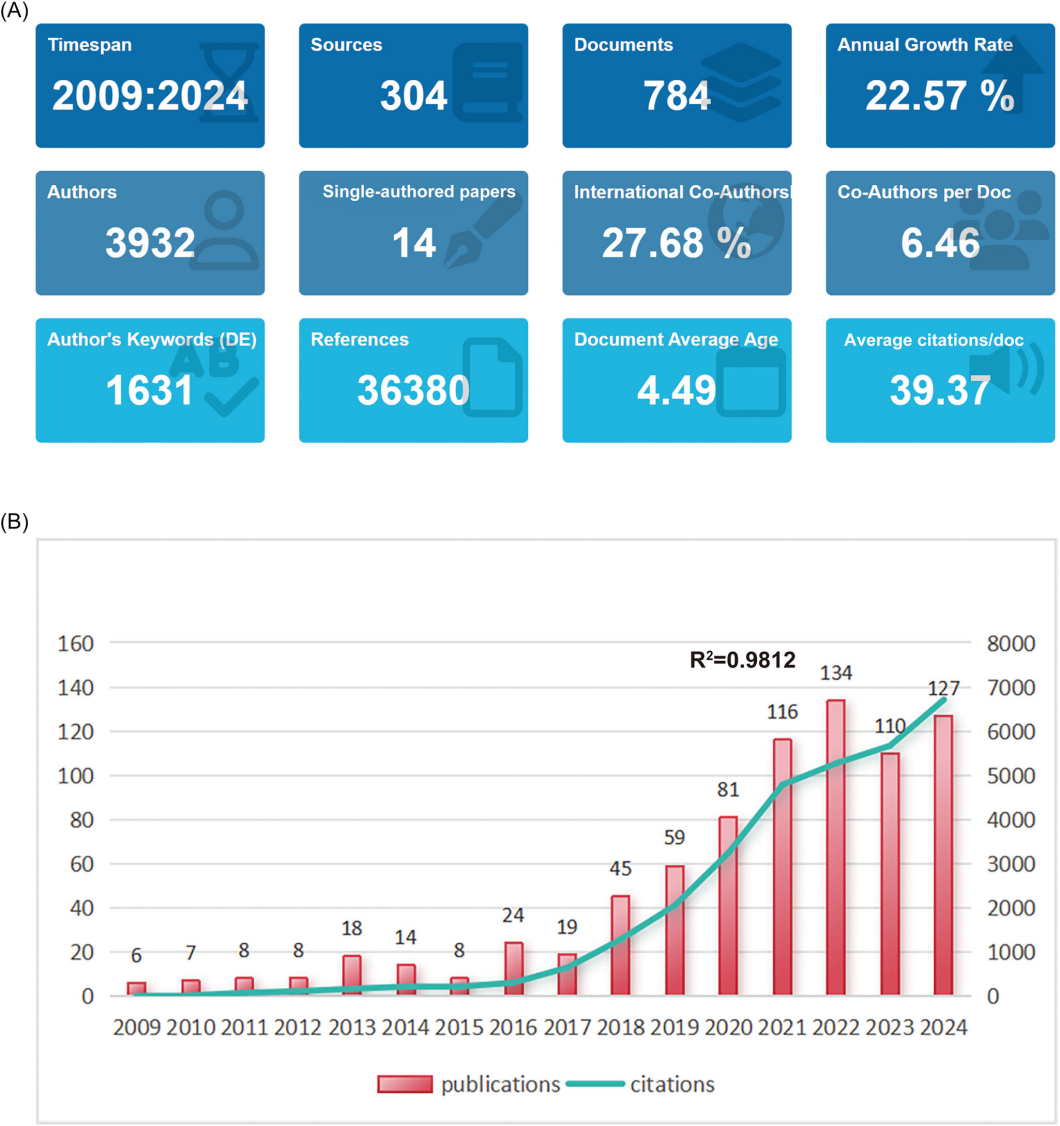
Overview of documents and trend of yearly publications and citations in the past 15 years. (A) Main information of all documents. (B) Times cited and publications over time. Red bars represent the number of papers related to LLPS and NDDs per year. The green line represents the trend‐fitted curve and the correlation coefficient (*R* ^2^) is displayed in the figure.

Figure 2A summarizes the main information of all included documents. The annual publication and citation trends in LLPS‐related NDD research from 2009 to 2024 are illustrated in Figure 2B. A total of 784 articles (see Supporting Information) were analyzed, showing a steady increase in annual publications observed over the 15‐year period. From 2009 to 2015, the field experienced slow growth (average annual growth rate: 15.1%), followed by exponential growth post‐2016 (average annual growth rate: 49.0%), aligning with Price's law of literature growth. By 2024, annual publications peaked at 112 articles, reflecting intensified interest in LLPS mechanisms and their implications in neurodegeneration. Citation counts mirrored this trend, with total citations surging from 5 in 2009 to 6712 by 2024 (see Supporting Information). The correlation coefficient (*R* ^2^ = 0.9612) between publication growth and the fitted exponential curve underscores the field's rapid expansion, driven by seminal discoveries linking LLPS to TDP‐43 aggregation in ALS [19] and subsequent explorations in AD, PD, and FTD [6].

### 3.2 Contribution of Countries/Regions and Collaboration Networks

**FIGURE 3:**
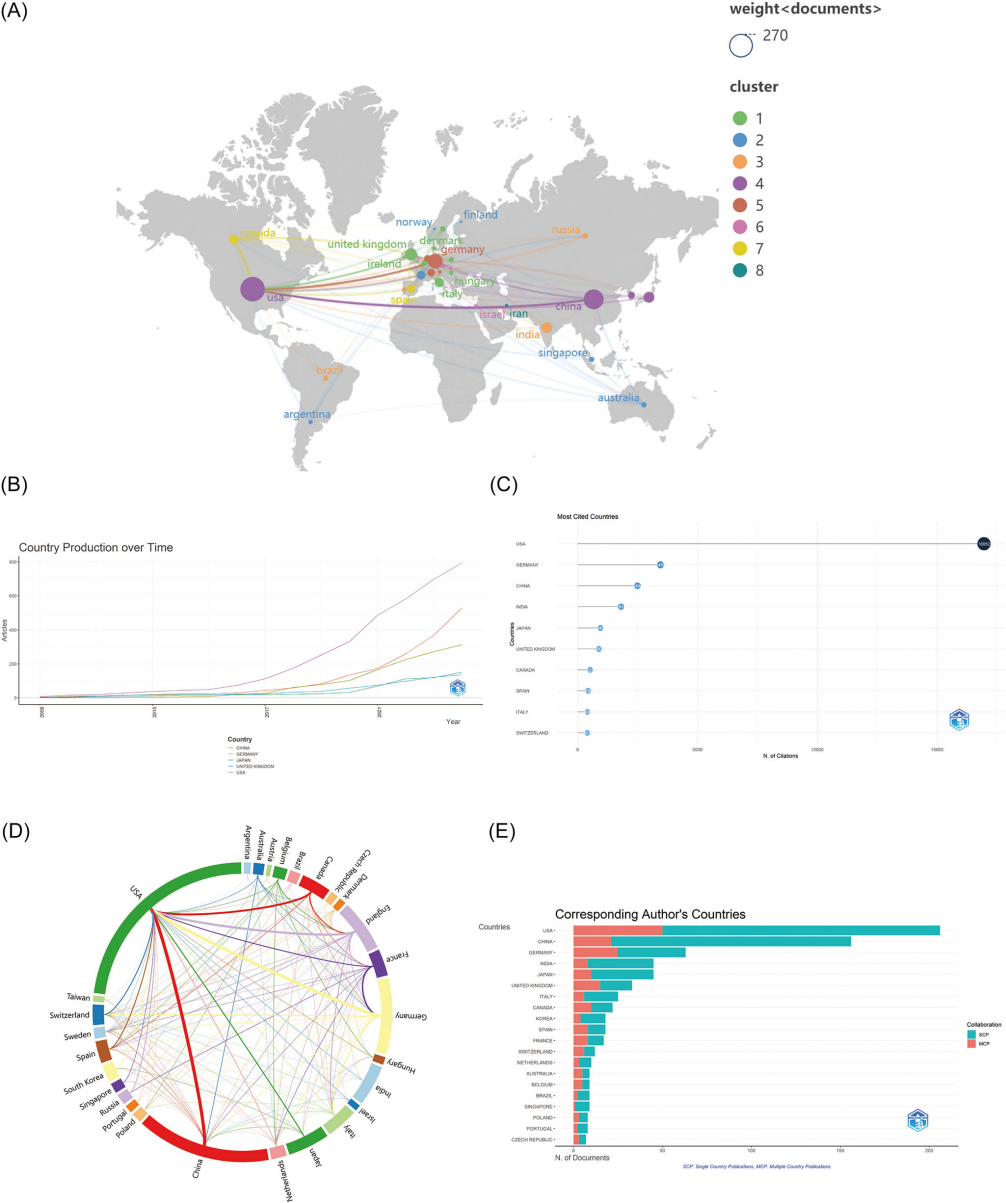
Contributions of countries/regions and collaboration among countries and regions. (A) Geographical distribution of global publications involved in the study of NDDs and LLPS. The width of the link lines represents the strength of collaboration between two countries. (B) Trends in annual publication volume of the top five countries/regions from 2007 to 2024. Lines show the cumulative number of publications. The *x*‐axis is year; the *y*‐axis is cumulative document count. (C) Top 10 countries/regions by citation impact (lollipop chart). Horizontal lines indicate total citation counts for publications affiliated with each country/region, with exact counts displayed within the terminal circles. (D) Chord diagram illustrating national research output and international collaborative networks. The outer arcs represent individual countries or regions, with arc length proportional to their total collaborative output. The connecting ribbons depict bilateral partnerships; the width of each ribbon indicates the strength of collaboration (number of co‐authored publications), and its color matches the originating arc. (E) Document distribution by country and collaboration type. The length of each blue bar segment within a country's bar indicates the number of documents published solely by authors from that country (SCP). The length of each red bar segment shows the number of documents published as a result of collaboration between authors from different countries (MCP). The MCP proportion indicates the extent of international collaboration.

Figure 3A delineates the geographical distribution and collaborative networks of LLPS–NDDs research. The United States dominated contributions (33.9% of publications), followed by China (21.6%) and Germany (12.2%). The top five countries/regions (including the United States, China, Germany, Japan, and the United Kingdom) with high annual publications showed a significant boost since 2017 (Figure 3B). These nations also exhibited the highest citation impact (Figure 3C), with the US leading at 16,952 citations. Collaborative networks (Figure 3D) revealed strong inter‐country partnerships, particularly between the United States and China, the United States and European nations (Germany, England, France), as well as the United States and Canada. The bar chart (single country publications [SCP]; multiple country publications [MCP]) provides a visual representation of the number of documents (publications) associated with corresponding authors from different countries (Figure 3E). The US leads with the highest number of documents in both SCP and MCP categories, indicating a strong presence in the field and significant engagement in international collaboration. China follows closely behind the United States, with a substantial number of documents in the MCP category, highlighting its growing role in international research partnerships. Other countries like Germany, India, and Japan also show considerable activity, with notable contributions in both SCP and MCP. The chart underscores the global nature of research in this field, with many countries participating in collaborative efforts (MCP), as well as individual research (SCP).

### 3.3 Contribution of Institutions and Collaboration Patterns

**FIGURE 4:**
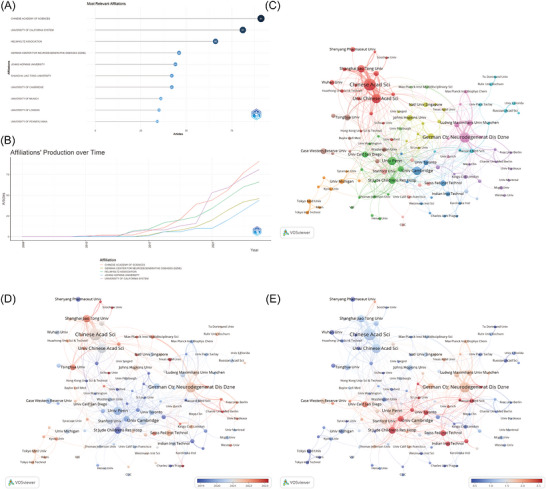
Contributions of institutions and collaboration among institutions. (A) The publication volume of top 10 most relevant affiliations. Horizontal lines indicate total citation counts for publications affiliated with each affiliation, with exact counts displayed within the terminal circles. (B) Trends in annual publication volume of the top five institutions from 2007 to 2024. Lines show the cumulative number of publications. The *x*‐axis is year; the *y*‐axis is cumulative document count. (C) The institutional cooperation network based on the VOSviewer (each point represents an institution, and the thicker the inter‐agency lines, the closer the cooperation). The size of each node is proportional to the total number of publications. Different colors represent distinct clusters, typically reflecting geographical or institutional affiliations. (D) The time‐overlay map illustrates the cooperation network among the top 100 most productive institutions. Node color indicates the APY, corresponding to the color gradient in the lower right corner. Blue signifies earlier appearance, whereas red indicates later appearance. (E) Collaborative maps based on institutional research impact. Red signifies higher research influence, whereas blue indicates relatively lower research influence.

Institutional contributions are mapped in Figure 4A,B. This scatter plot provides a clear overview of the top contributing affiliations in the field based on their publication output. The data highlight the following key points: The Chinese Academy of Sciences (CAS) is the most prolific institution, with 91 articles, indicating its leading role in the research field. The University of California (UC) system followed closely with 81 articles, which shows its significant contribution and impact. The Helmholtz Association, with 66 articles, has a strong presence in the field. The German Center for Neurodegenerative Diseases (DZNE), with 46 articles, is actively involved in research; and Johns Hopkins University, with 44 articles, is equally a key contributor in the field.

Figure 4A effectively illustrates the distribution of research efforts among different affiliations, providing valuable insights into the contributors to the field of phase separation and NDDs. The line graph (Figure 4B) offers a dynamic perspective on the research output of key affiliations in the field, revealing several trends. The CAS, represented by the red line, shows an upward trajectory, reflecting a consistent rise in published articles and highlighting its active contribution to the field over the years. Similarly, the UC system (purple line) shows consistent growth in publications, showcasing its active participation in advancing the field. The DZNE, depicted by the yellow line, also shows an upward trend, albeit at a slightly different rate, indicating its ongoing involvement and increasing publication output. The Helmholtz Association, marked by the green line, demonstrates a steady increase in the number of articles, underscoring its persistent efforts and growing influence in the field. Johns Hopkins University, shown by the blue line, presents a gradual ascent, signifying its continuous engagement in the research landscape.

Collaborative clusters create interdisciplinary networks, such as the China‐centric consortium involving CAS, University of Chinese Academy of Sciences (UCAS), and Shanghai Jiao Tong University; the multi‐institution UC system; and European institutions, including Ludwig‐Maximilians‐Universität München and DZNE (Figure 4C). In the overlay visualization map depicted in Figure 4D, institutions are represented by nodes, each color‐coded based on their average publication year (APY). As indicated by the color gradient in the lower right corner, institutions such as National University of Singapore, Texas A&M University, Soochow University, and Sichuan University are marked with a higher APY value, shown in red. This implies that a significant number of researchers from these institutions were recent entrants to this area of study. As illustrated in Figure 4E, the citation visualization map of institutions highlights those with a notably high citation rate in recent years in red. This implies that the research conducted by these organizations aligns closely with current research trends and focal points of interest. In contrast, institutions depicted in purple indicate a high citation rate in earlier years, with larger nodes signifying a greater impact; notable examples include the Harvard Medical School, St. Jude Children's Research Hospital, Swiss Federal Institute of Technology in Zurich, University of Cambridge, and University of Toronto.

### 3.4 Contribution of Authors and Co‐Citation Networks

**FIGURE 5:**
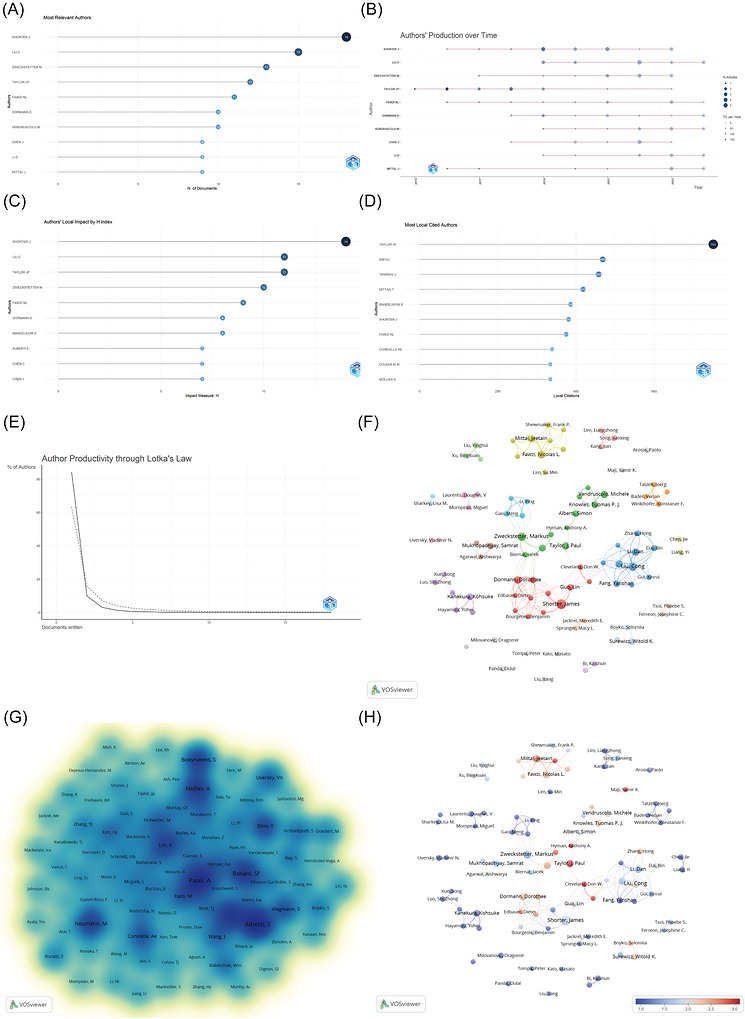
Contributions of authors and collaboration among them. (A) The publication volume of top 10 most relevant authors. (B) Publication output and citation impact of various authors over a span of year. Data point size shows annual article count (1–5+), and color intensity reflects total citations (0–150+), with darker colors for more citations. (C) Visualization of the local impact of authors based on their *h*‐index in the field of NNDs and LLPS. (D) The publication volume of top 10 most local cited authors. (E) The distribution of author productivity according to Lotka's law in the field of scientific literature. The solid line graph shows the actual distribution of author productivity. The dashed line likely represents the expected distribution according to Lotka's law. The solid line graph shows the actual distribution of author productivity. (F) Author co‐authorship analysis by VOSviewer. In the cluster density map, authors who frequently collaborate are grouped together in clusters colored identically. (G) The spectral density maps of the top 100 most co‐cited authors. The density of the map can help identify clusters of authors who are closely related in terms of their research interests and influence within the field. (H) Collaborative maps based on author research impact. Red signifies higher research influence, whereas blue indicates relatively lower research influence.

Author productivity, influence, and cooperation network are summarized in Figure 5. The document volume of top 10 most relevant authors is shown in Figure 5A. J. Shorter shows consistent publication activity with a notable peak in citations in 2019 (Figure 5A). J.P. Taylor also demonstrates a steady output with a significant number of citations received in 2016 (Figure 5B). C. Liu and M. Zweckstetter have a moderate number of publications and citations, indicating a steady level of research activity and impact in the field. Other authors, such as N.L. Fawzi, D. Dormann, M. Vendruscolo, J. Chen, D. Li, and J. Mittal, have varying levels of publication output and citation impact over the years. J. Shorter was identified as a leading contributor, with *h*‐indices of 14 (Figure 5C). J.P. Taylor was identified as the most local cited author (Figure 5D), which signifies that among the articles analyzed within the dataset, Taylor has received the highest number of citations from other authors within the same corpus. This suggests that Taylor's work is highly influential by being extensively referenced by peers in the specific domain of study. In the context of bibliometric analysis for SCI publication, this underscores Taylor's pivotal role in shaping current research trends and providing foundational work that other scholars build upon. The distribution follows Lotka's law, where a large number of authors publish a few documents, while a small number publish many, reflecting the highly skewed nature of scientific author productivity (Figure 5E). The dashed line likely represents the expected distribution according to Lotka's law.

Co‐authorship analysis (Figure 5F) identified different clusters, which are labeled by different colors, revealing distinct clusters centered around key authors, each with a specific research focus. By integrating the research contributions of the core authors identified in the co‐authorship network, several interesting insights emerge. The works centered around Shorter predominantly explore the dynamics of biomolecular condensates and the implications of TDP‐43 variants in neurodegenerative conditions. Meanwhile, the research associated with Taylor delves into the intricate relationship between tau and α‐synuclein phase separation, offering valuable perspectives on ALS pathogenesis. Dormann's research cluster reveals a concentrated effort on elucidating the pathological aggregation of TDP‐43 within NDDs, potentially uncovering novel therapeutic avenues. M. Zweckstetter leads a cluster that investigates the physicochemical properties of biomolecular condensates, contributing to our understanding of how phase separation events may lead to protein aggregation disorders. Collectively, these core authors and their respective clusters highlight the multifaceted nature of research in phase separation and NDDs, underscoring both the collaborative efforts and the diverse research trajectories within this scientific domain.

Co‐citation analysis (Figure 5G) highlighted foundational works by Brangwynne [6] on P granules and Patel [19] on FUS phase transitions, which remain pivotal to contemporary research. As shown in Figure 5H, the co‐citation author graph shows the top five most co‐cited authors were Alberti (citations = 375), Patel (citations = 337), Brangwynne (citations = 285), Banani (citations = 269), and Boeynaems (citations = 262).

### 3.5 Journals and Co‐Cited Journals Analysis

**FIGURE 6:**
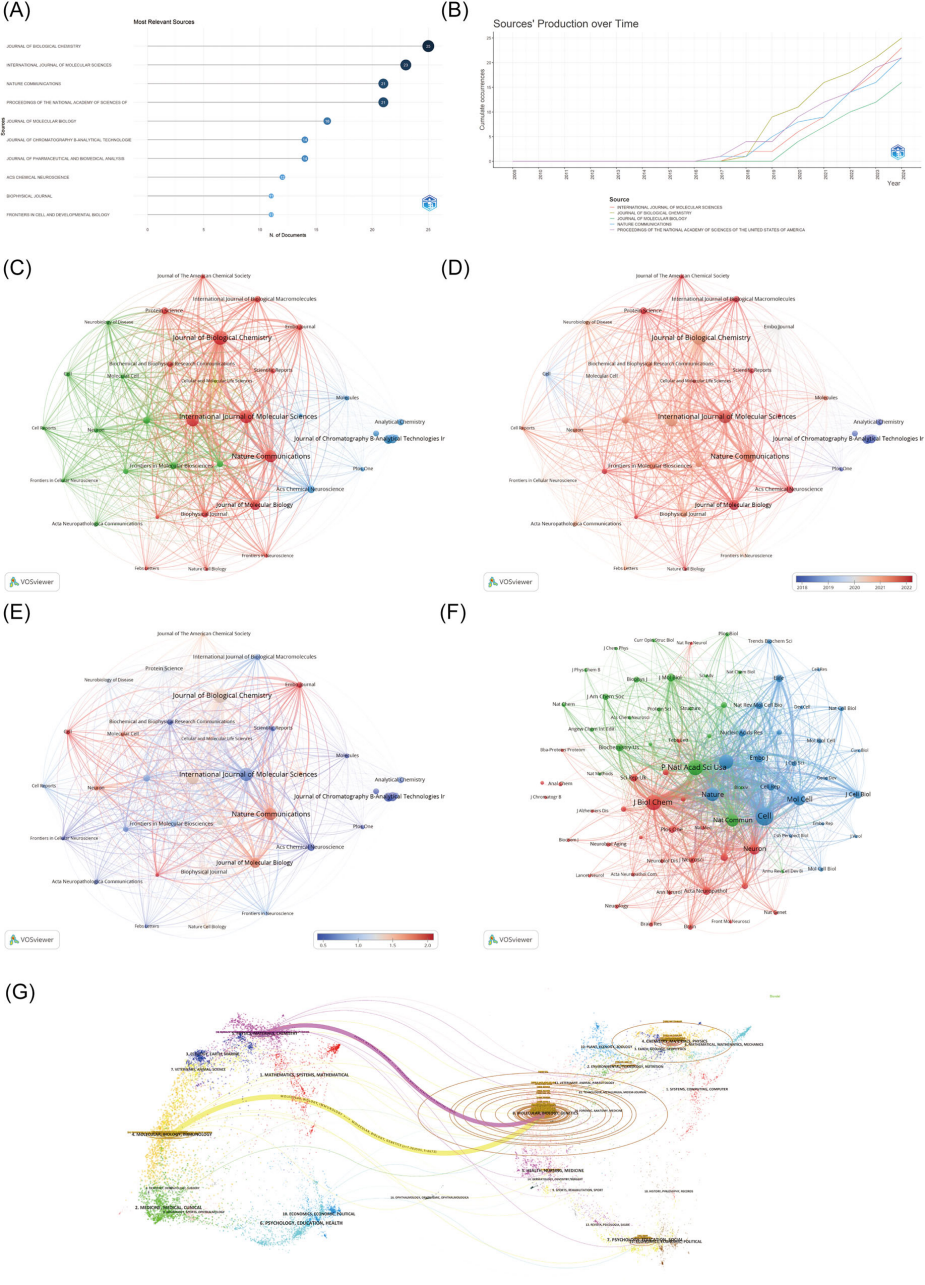
Contributions of journals and collaboration among them. (A) The publication volume of top 10 most relevant journals. (B) Trends in annual publication volume of the top five journals from 2009 to 2024. The *x*‐axis denotes years, and the *y*‐axis indicates publication counts. (C) The cooperation network among the most productive journals (co‐journal). Journals are denoted by individual nodes, sized according to their publication counts. Connecting lines depict citation relationships, with thicker lines indicating stronger citation ties. Different colors (green, blue, and red) highlight distinct clusters of frequently co‐cited journals, representing specific research themes. (D) The time‐overlay map illustrates the cooperation network among the most productive journals. Node color indicates the average appearing year (AAY), corresponding to the color gradient in the lower right corner. Blue signifies earlier appearance, whereas red indicates later appearance. (E) Collaborative maps based on institutional research impact. Red signifies higher research influence, whereas blue indicates relatively lower research influence. (F) The co‐citation network visualization map of journals. Each node represents a journal, and the lines connecting the nodes represent the co‐citation relationship. Journals are represented by nodes scaled according to their co‐citation frequency. Lines between nodes depict co‐citation relationships, with thickness reflecting the tie strength. Colors group frequently co‐cited journals into distinct clusters. (G) The dual‐map overlay of journals related to NDDs and LLPS. In the dual‐map visualization, citing journals are positioned to the left, whereas the cited journal is on the right. Citation relationships are highlighted by colored paths, where thicker lines denote the primary citation routes.

Figure 6 profiles the journal landscape. *Journal of Biological Chemistry, International Journal of Molecular Sciences*, and *Nature Communications* were the most relevant sources. The production of sources from the top five relevant journals (including *Journal of Biological Chemistry, International Journal of Molecular Sciences, Nature Communications, Journal of Molecular Biology*, and the *Proceedings of the National Academy of Sciences of the United States of America* (*PNAS*)) has shown a significantly active increase over time since 2019 and continued until 2024. As shown in Figure 6C, the journal citation graph showed that the five journals with the highest total link strength (TLS) were also the top five relevant journals. As shown in Figure 6D, the color gradient in the lower right corner indicates that journals such as *Nature Cell Biology*, *Scientific Reports*, *Nature Chemistry, Angewandte Chemie International Edition*, and *Journal of the American Chemical Society* are marked with a higher APY value, displayed in red. This suggests that a large proportion of publications from these journals have been contributed by recent entrants to the field. As depicted in Figure 6E, the citation visualization map of journals highlights those with a significantly high citation rate in recent years, also shown in red. This indicates that the research published by these journals is closely aligned with current research trends and areas of interest. Notable examples include *Cell, Nature Chemistry, EMBO Journal, Molecular Cell*, and *Nature Communications*. As shown in Figure 6F, a co‐citation map of journals was created using VOSviewer. The minimum number of citations was set at 150. A total of 78 journals met this threshold. The most frequently co‐cited journal was *Cell*, with a TLS of 359,357, followed by *PNAS, Journal of Biological Chemistry*, *Science*, and *Nature*. Furthermore, a dual‐map overlay of journals was constructed to visualize the subject distribution of academic journals (Figure 6G). The map displayed two distinct citation pathways, each represented by a different color.

### 3.6 Reference and Co‐Citation Analysis

**FIGURE 7:**
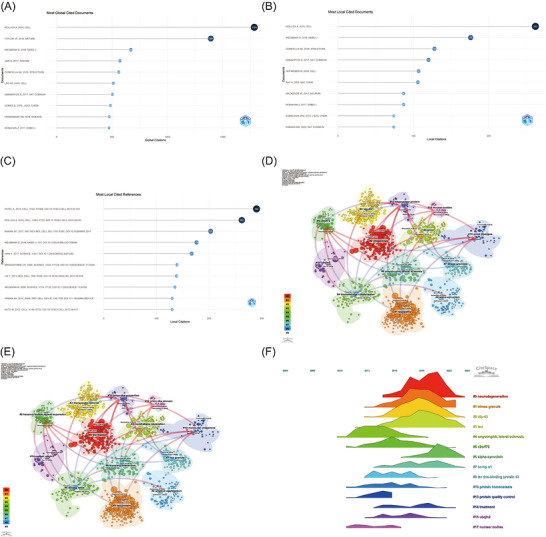
Analysis and visualization of references and co‐cited references. (A) The top 10 most global cited documents. (B) The top 10 most local cited documents. (C) The top 10 most local cited references. (D) Clustering of the co‐cited literature in the literature relationship network graph on the basis of keyword, with a total of 16 clusters. Node size corresponds to citation frequency, and edge thickness reflects co‐citation strength. Modularity‐based clusters are distinguished by distinct colors to highlight thematic diversity, with major groups labeled by their core keywords. (E) Clustering of the co‐cited literature in the literature relationship network graph on the basis of title, with a total of 16 clusters. (F) Reference co‐citation analysis visualized by the landscape view generated by CiteSpace with a time dimension. In this ridgeline timeline map, the starting point of each ridge on the horizontal axis indicates the time of first appearance. The height of the ridge is proportional to the number of citations of the reference.

Figure 7 analyzes citation dynamics. The most globally and locally cited document was Molliex et al. [20] (cited 1784 times), which indicates it is likely central to contemporary discussion (Figure 7A,B). Locally, Patel et al. [19] dominated (Figure 7B), linking LLPS to TDP‐43 aggregation, representing foundational or influential work external to our dataset.

Co‐citation clustering on the basis of keyword (Figure 7D) identified 16 thematic clusters. The development trend of the knowledge domain is mainly toward the right. By further plotting the timeline map, the evolution of each cluster can be more clearly observed. Clusters 13 (ribonuclear protein), 16 (nuclear bodies), 10 (prion diseases), and 8 (α‐synuclein) may represent emerging trends. If the titles of the articles are used for naming, different results will be obtained as shown in Figure 7E. Clusters 13 (prion‐like properties), 16 (prion‐like domain), 10 (molecular chaperone), and 8 (amyloid aggregation) may represent emerging trends. Reference co‐citation analysis visualized by the landscape view generated by CiteSpace with a time dimension (Figure 7F). By analyzing the changes in the peaks and valleys over different time periods, we can identify the emerging trends, the decline of certain research topics, and the shifts in research focus. “TDP‐42,” “tau,” and “SG” are topics that have been continuously and consistently hotly discussed, whereas “α‐synuclein” and “hnRNP a1” are emerging topics, demonstrating the research potential of the field.

**FIGURE 8:**
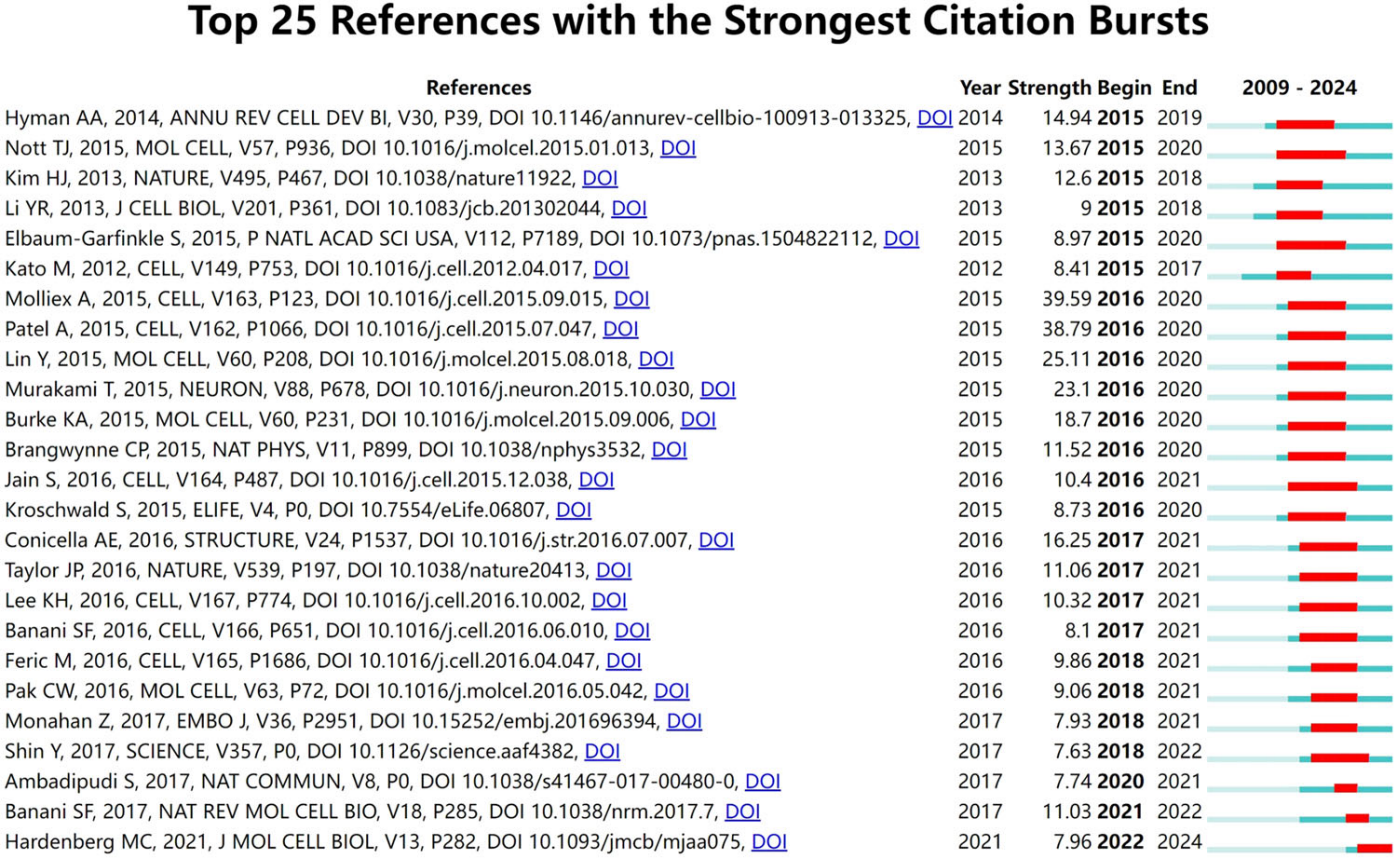
Top 25 highly burst‐cited literature. The blue line shows the timeline, and the red phase marks the period of peak citation activity.

The burst detection identifies sudden spikes in document references and significant developments in the field, such as new questions being raised or resolved within the literature. This method, developed by J. Kleinberg in CiteSpace, highlights periods of intense scholarly interest and advancement. Burst detection (Figure 8) highlighted emerging research, such as “Hardenberg M. C. 2021 (*J Mol Cell Biol*)” [60] (burst strength = 7.96, 2022–2024) and “Banani 2017 (*Nat Rev Mol Cell Bio*)” [4] (burst strength = 6.39, 2021–2022), as well as research with long‐term effects, such as “Elbaum‐Garfinkle S. 2015 (*PNAS*)” [61] (burst strength = 7.96, 2015–2020) and “Jain S. 2016 (*Cell*)” [62] (burst strength = 10.4, 2016–2021).

To further supplement the hierarchical analysis of citation impact, we categorized the top 25 references with the strongest citation bursts by document type (Figure 8). The analysis reveals a distribution of 18 original research articles (72%) and 7 comprehensive reviews or methodological guidelines (28%). This hierarchical ratio underscores a healthy scientific ecosystem where robust empirical breakthroughs are rapidly consolidated into conceptual frameworks.

Crucially, an in‐depth examination of these highly cited works delineates a clear evolutionary boundary between the field's core achievements and its emerging hotspots. The core achievements, primarily characterized by early‐burst original research (e.g., [19, 20]) and foundational reviews (e.g., [4, 6]), established the fundamental biophysical paradigm of the field. These seminal works definitively proved that intrinsically disordered regions (IDRs) in RNA‐binding proteins like FUS and TDP‐43 drive physiological LLPS, and that disease‐linked mutations fatally accelerate their liquid‐to‐solid phase transition into toxic aggregates [4, 6, 19, 20].

In contrast, the emerging hotspots, represented by recent citation bursts from 2018 onwards, mark a strategic expansion in both pathological scope and regulatory complexity. Although the core achievements narrowly focused on ALS‐linked proteins, emerging original studies have successfully pivoted toward the complex phase behavior of Tau in AD [25] and α‐synuclein in PD [26]. Furthermore, contemporary hotspots have shifted from merely describing the biophysical phenomenon to investigating its endogenous modulation. Recent highly cited works emphasize the regulatory roles of molecular chaperones and nuclear import receptors in preventing aberrant phase separation [28], alongside efforts to standardize rigorous experimental methodologies for condensate research [27]. This distinct transition highlights a field maturing from observational biophysics to mechanistic elucidation and therapeutic targeting.

### 3.7 Keyword Analysis

**FIGURE 9:**
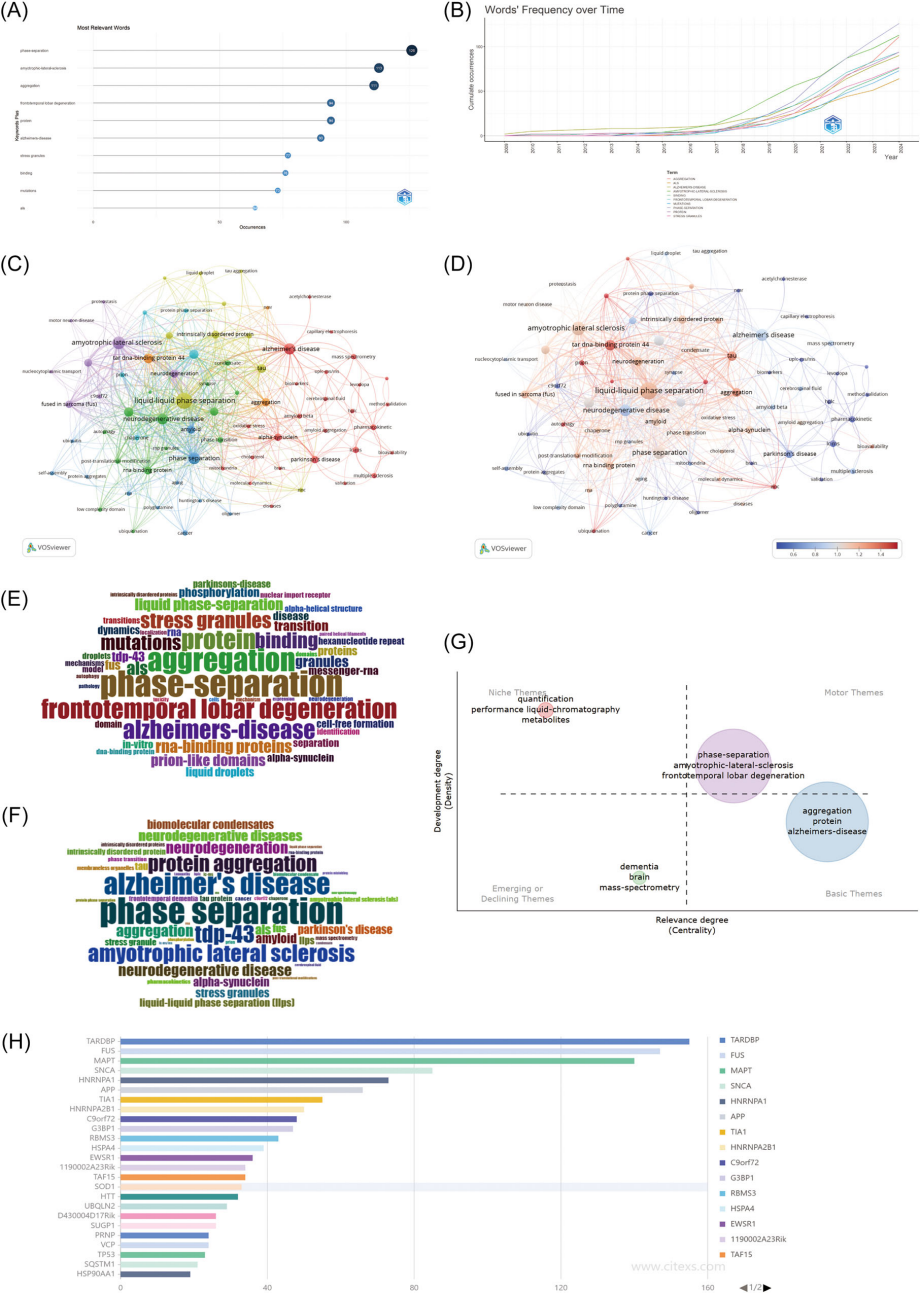
Keyword frequency and cluster analysis (A) The top 10 most relevant author keywords. (B) The frequency trend over time of top 10 author keywords with high frequency. (C) Network visualization map of author keywords co‐occurrence analysis. Within this map, keywords that have a close relationship are grouped into a cluster and are displayed in the same color. (D) Overlay visualization map of keywords co‐occurrence analysis. Red signifies higher research influence, whereas blue indicates relatively lower research influence. (E) Keywords plus word cloud based on bibliometrix package. (F) Author keywords word cloud based on bibliometrix package. (G) Research theme classification map. The map classifies research themes into four categories based on their development degree (density) and relevance degree (centrality). The four quadrants represent Niche Themes, Motor Themes, Emerging or Declining Themes, and Basic Themes, respectively. (H) A statistical analysis of associated genes is conducted, utilizing the BioBERT biomedical language representation model to analyze gene‐related entity words in the abstracts of articles.

The most relevant words are amyotrophic lateral sclerosis, frontotemporal lobar degeneration (FTLD), ALD, and SGs (Figure 9A), the cumulate occurrence of which was also increased gradually in recent years (Figure 9B). Keyword co‐occurrence analysis highlighted “TDP‐43,” “ALS,” “amyloid,” and “tau” as core research foci (Figure 9C). As illustrated in Figure 9D, the bibliometric network visualization of journal citations employs red highlighting to demarcate clusters of keywords exhibiting a surge in citation frequency over recent years. This pattern suggests that the core keywords—including “TDP‐43,” “tau,” “prion,” and “zinc,” and “autophagy”—demonstrate strong thematic alignment with emergent research frontiers and priority domains. The reference sources analyzed in this research produce a word cloud of keywords that are often used in articles (Figure 9D). The keywords that are often used are the word “FTLD,” “ALD,” “prion‐like domains,” “SGs,” “RNA‐binding proteins,” and “mutations.” The author keywords that are often used are the word “ALS,” “ALD,” “prion‐like domains,” “TDP‐43,” “SGs,” and “α‐synuclein.”

The strategy map of identified topics clustered by keywords plus. According to the keywords thematic map (Figure 9G), “phase‐separation,” “amyotrophic‐lateral‐sclerosis,” and “FTLD” were motor theme (upper right quadrant), which represents the important and well‐developed themes of the presbycusis research field. The basic themes (lower right quadrant) included “aggregation,” “protein,” and “ALD.” These aspects concern general topics that are transversal to different research areas of the field. Three specialized clusters with high developmental maturity yet lower centrality (categorized as niche themes within the upper left quadrant) were quantification, performance liquid‐chromatography, and metabolites. Concurrently, three nascent or diminishing thematic clusters were identified (characterized by weakly developed citation density and marginal positioning in the lower left quadrant), including dementia, brain, and mass‐spectrometry, the latter exhibiting proximity to foundational research domains based on temporal bibliometric indicators, signaling its potential transition toward a basic thematic status. The BioBERT biomedical speech representation model was used to mine and statistically analyze the entity words of genes in the abstracts of all articles. As shown in Figure 9H, TARDBP has the largest number of articles; FUS ranked second; MAPT ranked third.

**FIGURE 10:**
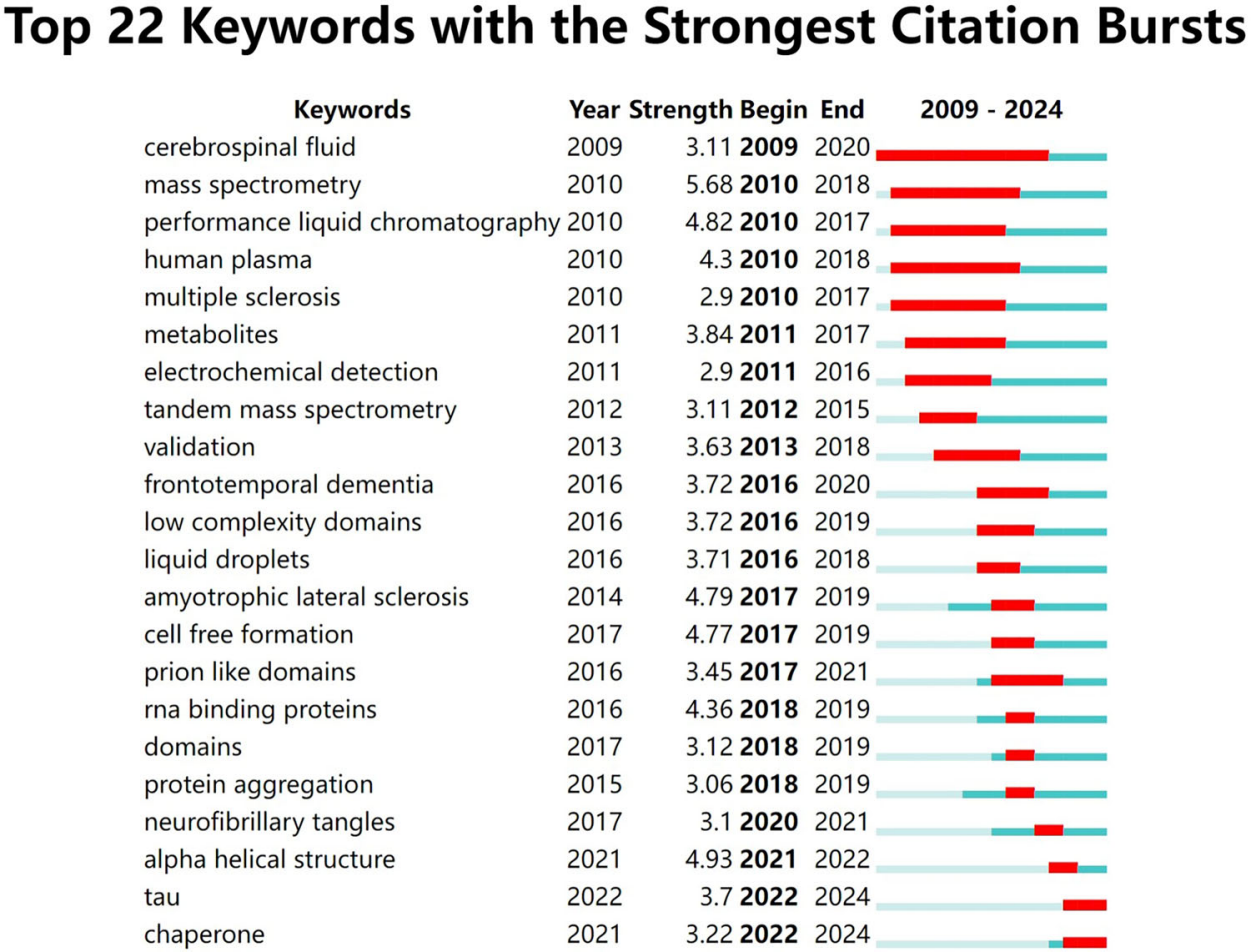
The top 22 keywords with the strongest citation bursts during 2007 to 2024. A blue line represents the timeline, whereas the red bars signify the burst period of keywords, covering their starting year, ending year, and the duration of the burst.

Burst detection revealed emerging trends, including “tau” (strength: 3.7, 2022–2024) and “chaperone” (strength: 3.2, 2021–2024) (Figure 10). Burst keywords like “cerebrospinal fluid” (strength: 3.11, 2009–2020) and “mass spectrometry” (strength: 5.68, 2010–2018) signify priorities with long‐term effects.

## 4 Discussion

### 4.1 General Trends and Cross‐Disciplinary Expansion

In recent years, LLPS has emerged as a new research hotspot in the field of NDDs. LLPS refers to the spontaneous separation of a homogeneous solution into two distinct phases, leading to the formation of membraneless compartments. This process plays a crucial role in the regulation of various cellular functions, and its dysregulation has been implicated in the pathogenesis of NDDs, where it can lead to the formation of toxic protein aggregates. This bibliometric analysis delineates the evolution of LLPS research in NDDs, capturing its transition from mechanistic discovery to therapeutic innovation. The analysis of 784 articles reveals a striking trajectory in research linking LLPS to NDDs, characterized by distinct growth phases and intensifying scholarly interest. The slow growth phase (2009–2015; 15.1% annual increase) likely reflects the foundational work establishing LLPS as a biologically relevant phenomenon. The inflection point in 2016 coincides with seminal studies linking LLPS to TDP‐43 aggregation in ALS [29], a discovery that catalyzed investigations across other NDDs, including AD, PD, and FTD. Post‐2016, the exponential increase in annual publications (49.0% growth rate) aligns perfectly with Price's law of scientific literature growth. The strong correlation (*R* ^2^ = 0.9612) between publication counts and the fitted exponential curve, culminating in a peak of 112 articles in 2024, underscores the field's rapid maturation. Concurrently, the surge in citations (from 5 in 2009 to 6712 in 2024) highlights the expanding influence and interdisciplinary convergence of biophysics, cell biology, and neuroscience.

Beyond neurodegeneration, the rapid expansion of LLPS research represents a broader cross‐disciplinary phenomenon, as evidenced by recent bibliometric analyses in oncology [30, 31]. Studies mapping phase separation in cancer have revealed similar exponential growth trajectories and geographic distribution patterns (e.g., the dominance of the United States and China). However, although NDD research primarily focuses on pathological protein aggregation and liquid‐to‐solid transitions, oncology bibliometrics highlight distinct mechanistic hotspots, such as LLPS‐driven transcriptional regulation, oncogene activation, and immune evasion within the tumor microenvironment [32]. The parallel evolution of these landscapes underscores how LLPS has fundamentally reshaped our understanding of disease pathogenesis across diverse biological contexts.

### 4.2 Drivers of Global Collaboration Landscape

The dynamic global landscape in LLPS research related to NDDs is characterized by concentrated contributions from leading nations and robust international collaboration. The United States emerged as the dominant force, accounting for 33.9% of total publications and leading in citation impact (16,952 citations), followed closely by China (21.6%) and Germany (12.2%). This triad of nations, alongside Japan and the United Kingdom, has driven the field's exponential growth since 2017. The sustained leadership of the United States and European institutions primarily reflects historical investments in biophysics and robust funding mechanisms, such as NIH grants and Horizon Europe projects. Conversely, China's rapid ascent mirrors its targeted strategic prioritization of neurodegenerative research, driven by policy initiatives and robust support from the National Natural Science Foundation of China (NSFC) [33]. However, the analysis also reveals geographic disparities, with limited contributions from Africa and South America, pointing to systemic inequities in global research infrastructure.

Collaborative networks and the analysis of SCP versus MCP further elucidate regional engagement. The United States dominated both categories, maintaining its dual role as a hub for independent innovation and global partnership. China's high MCP output highlights its strategic integration into international consortia. Transcontinental partnerships—particularly the United States–China and the United States–Europe alliances—have been pivotal in advancing mechanistic insights into LLPS‐driven aggregation. These collaborations synergize complementary strengths, coupling US expertise in molecular neuroscience with global advancements in biophysical tools (e.g., cryo‐electron microscopy [cryo‐EM] and super‐resolution imaging).

At the institutional level, the CAS emerged as the most prolific institution, reflecting the success of interdisciplinary neuroscience programs in China. Close behind, the UC system and Germany's Helmholtz Association demonstrated sustained productivity based on their established expertise. Furthermore, institutions such as the DZNE and Johns Hopkins University have solidified their roles as key contributors, aligning with their historical strengths in molecular pathology and clinical neurology.

### 4.3 Synthesis of Core Research Topics

To synthesize the bibliometric data into biological context, our analysis emphasizes that the overarching landscape of LLPS in NDDs is driven by three interconnected thematic pillars. (1) The ALS/FTD pathological hub: The most densely populated research topic centers on RNA‐binding proteins, predominantly TDP‐43 and FUS. Literature heavily emphasizes how mutations in their IDRs or chronic cellular stress disrupt RNA homeostasis, driving the irreversible maturation of physiological SGs into pathological cytoplasmic inclusions [34, 35]. (2) The expansion into AD and PD: A rapidly emerging topic is the phase behaviors of Tau and α‐synuclein. Recent high‐impact studies discussed in our network highlight that these proteins undergo LLPS prior to amyloidogenesis, and that specific microenvironmental triggers—such as metal ions, pH changes, and posttranslational modifications (PTMs) (e.g., hyperphosphorylation)—act as crucial catalysts for their liquid‐to‐solid phase transition and subsequent neurotoxicity [25, 26]. (3) Translational and therapeutic modulation: As the mechanistic understanding matures, the focal point of discussion in recent literature has shifted toward therapeutic interventions. This topic emphasizes the screening of small‐molecule modulators, molecular chaperones, and RNA‐based therapies aimed at stabilizing dynamic liquid condensates, dissolving aberrant aggregates, or rescuing nuclear‐cytoplasmic transport defects [36, 37]. By mapping these core topics, this study not only quantifies publication trends but also delineates the distinct biological trajectories shaping the future of neurodegeneration research.

### 4.4 Spatiotemporal Evolution of Biological Hotspots

A joint analysis of institutional engagement, author collaboration networks, keyword bursts, and journal co‐citations reveals that the 15‐year evolution of the field follows a distinct three‐phase spatiotemporal trajectory.

1. Early discovery and paradigm foundation (2009–2017): Initially, research was predominantly driven by a concentrated network of pioneering institutions (e.g., St. Jude Children's Research Hospital, Max Planck Institute) and authors (e.g., Taylor, Shorter, and Alberti). Their highly cited foundational works established the fundamental liquid‐to‐solid transition model in ALS and FTLD. Co‐citation networks underscore seminal studies by Brangwynne et al. [5] on biomolecular condensates, alongside Molliex et al. [20] and Patel et al. [19], which bridged LLPS with TDP‐43 cytoplasmic mislocalization and aggregation. During this phase, the core focus remained on RNA‐binding proteins and their IDRs, solidifying LLPS as a mechanistic scaffold for proteinopathy [18, 38, 39, 40].
2. Mechanistic expansion and biological universality (2018–2022): As the collaborative network expanded globally—marked by the rapid ascent of comprehensive hubs like the CAS and the UCAS—the research paradigm shifted. The citation hotspots diversified from purely ALS models to the phase behavior of tau and α‐synuclein in AD and PD. Our BioBERT analysis confirms *TARDBP*, *MAPT*, and *FUS* as the most frequently studied genes during this expansion, reflecting their central roles across varied pathologies [41, 42, 43]. This phase revealed how PTMs regulate condensate homeostasis. PTMs (e.g., hyperphosphorylation and acetylation) are no longer viewed merely as markers of pathology, but as dynamic rheostats and molecular switches dictating the irreversible maturation of droplets into neurotoxic states [44, 45]. The dual‐map overlay of journals mirrors this transition, showing strong citation linkages between biophysics/molecular journals (e.g., *Cell* and *Nature Communications*) and clinical neurology (e.g., *Lancet Neurology*). Studies like Wegmann et al. [25] and Guillén‐Boixet et al. [3] exemplify this by demonstrating tau LLPS in patient‐derived neurons and SG dysregulation in FTD, respectively.
3. Recent translational frontiers (2023–2024): The contemporary landscape is characterized by terminal citation bursts for keywords such as “tau,” “molecular chaperone,” and “small molecule,” signaling a transition toward “phase engineering.” The rapid rise of “tau” as an emerging hotspot is rooted in the shifting paradigm of AD research, where the focus has moved beyond amyloid‐β to the liquid‐to‐solid transition of tau protein [45]. The rapid rise of “chaperone” highlights a shift from describing pathological phenomena to exploring cellular defense mechanisms. The latest research repositions molecular chaperones (e.g., HSP70 and HSPB8) as active phase modulators that maintain condensate fluidity and prevent aberrant liquid‐to‐solid transitions [46, 47, 48]. In the past 2 years, groundbreaking evidence has shown that the specific recruitment of chaperones to SGs or pathological droplets can prevent the aberrant liquid‐to‐solid transition, offering a potential therapeutic window for rescuing proteostasis in ALS and PD [49]. The convergence of “tau” and “chaperone” in recent clusters underscores a burgeoning research direction: leveraging the cell's innate proteostatic machinery to therapeutically modulate the phase behavior of intrinsically disordered proteins. Simultaneously, translational efforts are increasingly targeting upstream LLPS regulators. Researchers are actively deploying small‐molecule interventions designed to either regulate condensate material properties or halt the condensation‐driven aggregation of α‐synuclein [50, 51].

By integrating these multidimensional metrics, we identify three primary drivers for this paradigm shift. First, the realization of biological universality: The recognition that IDR‐driven phase transitions are highly conserved across diverse neurodegeneration‐associated proteins [25]. Second, technological breakthroughs: Advanced interrogation tools, such as optoDroplets and cryo‐EM, enabled precise structural resolution of pathological condensates [52, 53]. Finally, a translational imperative: As the fundamental biophysical principles of phase separation have matured, research priorities have strategically pivoted toward therapeutic intervention. Contemporary efforts are increasingly focused on deploying synthetic small‐molecule modulators to regulate condensate dynamics and leveraging the innate proteostasis machinery to prevent or dissolve aberrant, disease‐linked aggregates [29, 54]. Ultimately, this demonstrates that the structural expansion of the global collaboration network is fundamentally linked to the field's transition from in vitro biophysics to next‐generation clinical interventions.

### 4.5 Future Perspectives: Toward Phase Engineering

By synthesizing the results of keyword clustering and citation burst analysis, we identify three core research directions that are poised to dominate the landscape of LLPS in NDDs over the next 3–5 years. First, the field is transitioning from a descriptive phase to a precise regulatory phase, centered on the role of PTMs and molecular chaperones. As evidenced by the 2023–2024 burst in keywords like “chaperone” and “tau,” future efforts will increasingly focus on how endogenous proteostatic machinery (e.g., HSP70/DNAJB1 complexes) selectively preserves the fluidity of biomolecular condensates or actively reverses pathological liquid‐to‐solid transitions [46, 48]. Elucidating a PTM “code”—the specific combinations of phosphorylation, acetylation, and methylation that dictate protein phase behavior—would be essential for developing high‐resolution mechanistic models of disease progression [45]. Second, the integration of multi‐pathology “co‐phase separation” represents a burgeoning frontier. While early research focused on isolated proteins, the next few years will likely see a surge in studies investigating the synergistic LLPS behavior between different pathological proteins—such as the synchronized electrostatic coacervation and co‐aggregation of tau and α‐synuclein. Understanding how diverse MLOs crosstalk and how one pathological condensate facilitates the seeding of another will provide a more holistic view of mixed‐proteinopathies, which are common in clinical settings [55]. Third, the translational paradigm is shifting toward the high‐throughput screening of phase‐modifying small molecules. Our joint analysis of recent hotspots underscores that the therapeutic targeting of LLPS is moving from in vitro proof‐of‐concept to AI‐augmented drug discovery. The upcoming research cycle will prioritize molecules—often termed condensate‐modifying drugs (c‐mods)—that do not simply dissolve all condensates, which could impair physiological functions, but instead specifically stabilize the functional state of droplets or regulate aberrant material property transitions [49, 56]. These directions, fueled by technological advances, will likely transform LLPS from a biophysical curiosity into a foundational platform for next‐generation NDD modifiers.

### 4.6 Paradigm Shift to “Condensatopathy”

Beyond the discrete metrics of publications and citations, this bibliometric landscape uncovers a compelling conceptual evolution: the transition of neurodegenerative research from a static proteinopathy paradigm to a dynamic condensatopathy framework. Our synthesis of a decade‐long data trajectory reveals that the core insight of the field is no longer the mere presence of aggregates, but the aberrant life cycle of biomolecular condensates. The shift in research hotspots—from early identification of TDP‐43 droplets to the current focus on PTMs and molecular chaperones—reflects a deepening understanding that phase separation serves as the primary “pathological sieve” [34, 57]. In this framework, LLPS acts as a double‐edged sword: It provides essential spatiotemporal compartmentalization for RNA metabolism under physiological conditions, but its dysregulation creates crucibles for amyloid nucleation. Consequently, the most profound insight for future research is that therapeutic success will likely depend on condensate‐modifying therapeutics or phase‐targeted interventions—not merely attempting to clear final aggregates, but restoring the dynamic homeostasis of the cellular condensate landscape [58]. This paradigm shift, mapped by our bibliometric analysis, suggests that strategically disrupting the pathologic liquid‐to‐solid phase transition represents the most promising upstream intervention for the next generation of NDD‐modifying therapies [57, 59].

## 5 Conclusion

This study provides a comprehensive 15‐year (2009–2024) bibliometric landscape of LLPS in NDDs, mapping its evolution from a niche biophysical observation to a central pillar of neurobiology. Our analysis reveals that the research landscape has entered a stage of exponential growth, characterized by the global dominance of the United States and China and the maturation of highly interconnected institutional networks. The most profound insight derived from this bibliometric synthesis is the ongoing paradigm shift from static proteinopathy to dynamic condensatopathy. While early research hotspots focused on the identification of TDP‐43 and FUS droplets in ALS models, current trends emphasize the universal role of the liquid‐to‐solid phase transition across diverse pathologies, including tauopathies in AD and α‐synuclein aggregation in PD. We conclude that LLPS serves as the primary pathological sieve, where the disruption of IDR dynamics—driven by site‐specific PTMs and environmental stress—triggers the formation of irreversible, neurotoxic inclusions. Looking forward, the core research directions for the next few years will likely pivot toward phase engineering. This includes the strategic deployment of molecular chaperones (e.g., HSP70/DNAJB1) to maintain condensate fluidity and the AI‐driven discovery of small‐molecule modulators designed to stabilize functional liquid states. In summary, the intellectual architecture of this field is transitioning from fundamental mechanistic mapping to active therapeutic intervention. By targeting the dysregulated phase behavior of proteins, the next generation of disease‐modifying therapies may finally address the upstream origins of neurodegeneration.

### 5.1 Limitations

Data scope: Reliance on SCI‐indexed journals excludes preprints and regionally influential studies (e.g., non‐English publications on LLPS in Lewy body dementia), potentially skewing geographic and thematic representation.

Temporal bias: Recent breakthroughs (e.g., m^1^A–TDP‐43 interactions and APEX2‐based condensate mapping) may be underrepresented due to citation lag, particularly for 2024 publications.

Methodological constraints: Collaborative networks reflect formal co‐authorships but overlook informal knowledge exchanges, such as unpublished data shared in consortia like the European DZNE.

Clinical translation gaps: Although in vitro and animal models dominate LLPS research (e.g., *Caenorhabditis elegans* studies), human‐centric validation remains limited, as seen in conflicting biomarker studies of α‐synuclein in PD.

These limitations highlight the need for inclusive, technology‐driven frameworks to advance LLPS–NDD research toward equitable and clinically actionable outcomes.

## Author Contributions

Conceptualization: Chanyuan Zhang, Siyao Chen, and Haixia Fan. Methodology: Haixia Fan. Software: Chanyuan Zhang. Validation: Chanyuan Zhang and Lingyan Zhou. Formal analysis: Chanyuan Zhang. Investigation: He Zhao. Data curation: Yan Wang. Writing – original draft preparation: Chanyuan Zhang and Siyao Chen. Writing – review and editing: Haixia Fan. Visualization: Yan Sun. Supervision: Yan Sun. Funding acquisition: Yan Sun. All authors have read and agreed to the published version of the manuscript.

## Funding

This work was supported in part by the National Natural Science Foundation of China (#82371153 and #82571317), the Natural Science Foundation of Shandong Province (#ZR2022QH073 and #ZR2025MS1188), and the Natural Science Foundation of Yantai (#ZR2025LZ044) (to Y.S.).

## Conflicts of Interest

The authors declare no conflicts of interest.

## Supporting information




**Supplementary Materials**: nyas70311‐sup‐0001‐SuppMat.xls

**Supplementary Materials**: nyas70311‐sup‐0002‐SuppMat.xlsx
